# A Monoclonal Antibody Combination against both Serotypes A and B Botulinum Toxin Prevents Inhalational Botulism in a Guinea Pig Model

**DOI:** 10.3390/toxins13010031

**Published:** 2021-01-05

**Authors:** Doris M. Snow, Ronald R. Cobb, Juan Martinez, Isaac Finger-Baker, Laura Collins, Sara Terpening, Emily S. Syar, Nancy Niemuth, Dean Kobs, Roy Barnewall, Shauna Farr-Jones, James D. Marks, Milan T. Tomic

**Affiliations:** 1 Ology Bioservices, 13200 NW, Nano Ct, Alachua, FL 32615, USA; doris.snow@ologybio.com (D.M.S.); ron.cobb@ologybio.com (R.R.C.); juan.martinez@ologybio.com (J.M.); isaac.finger@ologybio.com (I.F.-B.); laura.collins@ologybio.com (L.C.); sara.terpening@ologybio.com (S.T.); 2 Battelle Biomedical Research Center, West Jefferson, Columbus, OH 43162, USA; syare@battelle.org (E.S.S.); niemuthn@battelle.org (N.N.); kobsd@battelle.org (D.K.); barnewallr@battelle.org (R.B.); 3 Department of Anesthesia and Perioperative Care, University of California, 1001 Potrero Ave., San Francisco, CA 94110, USA; shauna.farr-jones@ucsf.edu (S.F.-J.); jim.marks@ucsf.edu (J.D.M.); 4 Research and Development, Ology Bioservices, Inc., 2061 Challenger Dr., Alameda, CA 94501, USA

**Keywords:** botulinum neurotoxin, aerosol, monoclonal antibody, guinea pig inhalation model, oligoclonal antibody, botulism

## Abstract

Botulinum neurotoxins (BoNT) are extremely potent and can induce respiratory failure, requiring long-term intensive care to prevent death. Recombinant monoclonal antibodies (mAbs) hold considerable promise as BoNT therapeutics and prophylactics. In contrast, equine antitoxin cannot be used prophylactically and has a short half-life. Two three-mAb combinations are in development that specifically neutralize BoNT serotype A (BoNT/A) and B (BoNT/B). The three-mAb combinations addressing a single serotype provided pre-exposure prophylaxis in the guinea pig inhalation model. A lyophilized co-formulation of six mAbs, designated G03-52-01, that addresses both A and B serotypes is in development. Here, we investigated the efficacy of G03-52-01 to protect guinea pigs against an aerosol exposure challenge of BoNT/A1 or BoNT/B1. Previously, it was found that each antibody demonstrated a dose-dependent exposure and reached maximum circulating concentrations within 48 h after intramuscular (IM) or intravenous (IV) injection. Here we show that G03-52-01, in a single IM injection of G03-52-01 administered 48 h pre-exposure, protected guinea pigs against an aerosol challenge of up to 238 LD_50_s of BoNT/A1 and 191 LD_50_s of BoNT/B1. These data suggest that a single IM administration of G03-52-01 provides pre-exposure prophylaxis against botulism from an aerosol exposure of BoNT/A1 or BoNT/B1.

## 1. Introduction

Botulism can be fatal if untreated and is caused by exposure to any one of the botulinum neurotoxins (BoNTs). BoNTs can be classified into at least seven serotypes (A–G) [1] defined immunologically by the inability of IgG antibodies that neutralize one serotype to neutralize the other serotypes [2]. BoNT serotypes A, B, E, and F cause the disease botulism in humans [3,4], with the majority of cases within the United States being caused by BoNT/A and B [4]. Worldwide, most botulism cases are caused by BoNT/A, B, and E, with BoNT/F being associated with 1% of food poisoning-related cases of botulism intoxication [5,6,7,8]. BoNTs are generally regarded as the most potent of all biological poisons [9,10].

BoNT is the agent responsible for botulism [5], a disease with four naturally occurring etiologies: foodborne, wound, infant botulism, and adult intestinal botulism. A fifth etiology is iatrogenic botulism [6,7]. Botulism can also be caused by BoNT inhalation [8,9,10]. Each of these exposures results in similar clinical symptoms, including symmetrical cranial nerve palsies followed by descending, symmetric, flaccid muscle paralysis of voluntary muscles that may progress to respiratory compromise, which may require 2 to 8 weeks on mechanical ventilator support, or death [3,11]. There is considerable published literature on the BoNT mode of action [12]. For example, the mechanism of BoNT poisoning by the inhalational route has been addressed and studies have demonstrated that passive immunization of mice with antibodies can prolong survival after aerosolized toxin exposure [13,14]. Additionally, antibodies have been shown to reduce the levels of circulating toxin [13]. Once the toxin reaches general circulation, the combination of three circulating antibodies effectively neutralizes the toxin [15,16], preventing BoNT poisoning.

BoNT molecules are the deadliest toxins known, and there are a couple of requirements to BoNT poisoning—the toxin has to be expressed by a clostridial species and it needs to have significant demonstrable toxicity [12,17]. Genomic analysis can reveal broad diversity in BoNT-producing clostridial strains [18], and bioinformatics methods can identify BoNT-like molecules and may help explain the evolution of BoNT [19,20]. The relevance of the newly discovered or engineered BoNT-like molecules, however, is not clear. For example, BoNT/X, which was found in a clostridial strain expressing BoNT/B2 [21], had to be modified for expression and study, and the natural expression of this toxin has not been demonstrated. Additionally, the potency of these novel BoNT-like toxins is unclear; for example, it was reported that a recombinant derivative of BoNT/X required 0.5 ug to show effect on a mouse footpad assay, which is greater than 1000-fold more than required for mouse poisoning by BoNT/A [17].

Some BoNTs are an approved medication for the treatment of a variety of conditions [22,23] and have been widely used and available for cosmetic applications [24]. BoNTs are also being studied for their ability to act as transepithelial carriers to create oral and inhalation drugs [25] and as neuronal carriers for delivery to cholinergic nerves [26]. Because these extremely potent and commercially used toxins cause botulism, BoNTs have the potential to be used in acts of bioterrorism and biological warfare. Therefore, they are classified as Tier 1 biothreat agents by the Federal Select Agent Program of the Centers for Disease Control and Prevention (CDC) [27]. Increased medicinal use of BoNTs and their legal and illicit manufacture makes BoNTs readily available to those of ill intent [28,29]. 

Currently, there is no United States Food and Drug Administration (FDA)-licensed BoNT vaccine available. An investigational vaccine against BoNT serotypes A–E was distributed by the CDC for laboratory workers and the military. It was withdrawn from clinical use in 2011 due to the decrease in potency [30,31]. Several recombinant BoNT vaccines are under development, with the most advanced a bivalent serotype A and B based on the HC domain (rBV A/B) and expressed from *Pichia pastoris* [32,33]. The rBV A/B is being developed specifically for the Department of Defense’s Joint Vaccine Acquisition Program to protect warfighters against inhalational botulism [34]. The rBV A/B has undergone two Phase 1 and one Phase 2 clinical studies [34]. In the Phase 1 trials, more than 80% of patients developed detectable neutralizing antibody titers against BoNT/A and BoNT/B, and longer vaccination schedules demonstrated increased neutralizing antibody titers compared to shorter schedules. The Phase 2 trial demonstrated neutralizing antibodies up to 182 days, but the duration of protection was not reported. As of October 2020, however, there is not a licensed vaccine approved for this indication. Furthermore, a concern for vaccines to BoNT is that treatment with BoNT for conditions, including spasticity, would be precluded in those vaccinated.

An alternative to vaccination is the prophylactic administration of neutralizing antibodies or antitoxins that would provide immediate immunity to all of the dosed subjects. These antibodies or antitoxins would need to be made in adequate quantities and have half-lives of weeks to provide sufficient protection against intoxication. Currently, two antitoxins have been approved for the treatment of botulism. These include equine-derived heptavalent botulinum antitoxin (BAT)^®^ [35] and human botulism immune globulin (BIG-IV) [36]. BAT is used to treat non-infant botulism and is a Fab’2 antibody derived from immunized horses. BAT treatment is associated with hypersensitivity and has a very short half-life (7.5–34.2 h depending on serotype), which precludes BAT use to prevent botulism since protective serum levels of antibody are short lived, on the order of days [35]. BIG-IV is produced by plasmapheresis of immunized human laboratory workers, and the small quantities produced preclude its use for large-scale passive immunization of adults [11]. Thus, neither BAT nor BIG-IV are vaccine alternatives. Human or humanized antibodies with high potency would allow for immediate overall effectiveness, increased safety and reproducibility, lower overall doses, and longer duration of protection.

Here we tested a potent human immunoglobulin (IgG) monoclonal antibody (mAb)-based drug product, G03-52-01, composed of six co-formulated IgG mAbs that bind to non-overlapping epitopes of BoNT/A [15] and BoNT/B [37,38]. The BoNT/A and BoNT/B antitoxins comprising G03-52-01 (NTM-1631 and NTM-1632) have both completed Phase 1 testing in humans without serious adverse events [39] and unpublished results. These drugs are an equimolar combination of three antibodies and have also been shown to be efficacious in inhalational botulism models in guinea pigs [38]. To determine the ability of BoNT/A and BoNT/B mAb cocktails when used in combination as an alternative to vaccination for the prevention of inhalational botulism, we evaluated the ability of G03-52-01 administered by the intravenous (IV) or intramuscular (IM) route, using an aerosol delivery model in guinea pigs. The goal of the first study was to evaluate the lowest dose of G03-52-01 that still provides protection. In Study 1, animals dosed with as little as 0.003 mg of total antibodies were protected (9 of 10 animals survived) against 31 guinea pig median lethal doses (GPLD_50_s) of BoNT/A1 and 46 GPLD_50_s of BoNT/B1. The goal of Study 2 was to evaluate lower doses of G03-52-01 and evaluate its ability to protect animals from aerosol challenge in order to determine breakthrough of protection. In Study 2, all of the animals challenged with greater than 169 GPLD_50_s of BoNT/A1 succumbed when dosed with less than 0.03 mg of total antibodies. Only two of ten animals challenged with at least 191 GPLD_50_s of BoNT/B1 toxin survived when given a dose of 0.025 mg of total antibodies, and all animals that were given a lower dose did not survive the increased challenge dose. This study demonstrated a dose–response relationship and identified the lowest dose that prevents death. The objective of these studies was to determine the efficacy of G03-52-01 against an inhalation challenge of BoNT/A1 or BoNT/B1 following a single IM injection. The amount of toxin neutralization afforded by the antibodies was assessed using the mouse neutralization assay (MNA).

## 2. Results

### 2.1. Evaluation of Dose and Route of Administration of G03-52-01 Neutralizing Antibody Concentration (NAC) in Guinea Pigs

The concentration of each of the six antibodies that comprise G03-52-01 demonstrated peak or near-peak mAb serum concentrations at 48 h after IM injection in guinea pigs. Antibody concentrations of each of the antibodies could be detected up to 28 days post-IM injection [38]. Further, NAC was found up to 14 days post-IM injections, with doses as low as 0.06 mg/animal of anti-BoNT/A and 1.5 mg of anti-BoNT/B [38].

**Figure 1:**
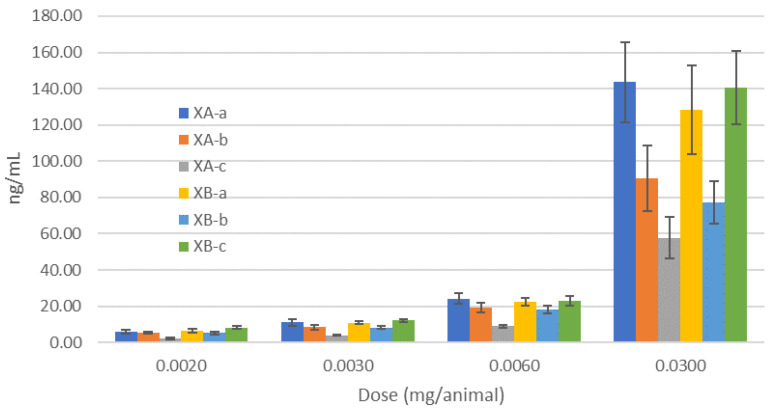
Serum concentration of individual antibodies in guinea pigs, 48 h after dosing IM in first challenge experiment. Mean levels of antibodies ± standard deviation are shown. Animals were dosed with the designated levels of antibodies. Serum samples were taken prior to dosing to ensure the antibodies were indeed in circulation in the animals prior to challenge.

Three guinea pigs were terminally bled on the day of the BoNT challenge (approximately 48 h after IM administration of G03-52-01), and NAC was measured. Serum concentrations of each antibody were shown to be similar to previous studies (Figure 1) [38]. The MNAs from guinea pigs treated with total G03-52-01 concentrations of 0.03 mg/animal, IM injection (Group 2) resulted in a NAC value of 0.11 U/mL for serotype A. We were unable to measure XA-b in this experiment due to the instability of the antibody-specific BoNT domain; nevertheless, protection from the BoNT challenge indicated that it was present. The geometric mean NAC for the groups that received 0.002, 0.003, or 0.006 mg/animal (Groups 5, 4, and 3, Table 1) were below the lower level of quantitation (LLOQ) for serotype A. For serotype B, guinea pigs administered with total G03-52-01 concentrations of 0.003, 0.006 or 0.03 mg/animal (Groups 9, 8 and 7, Table 1) all had NACs above the LLOQ with geometric mean NACs of 0.015, 0.097, and 0.46 U/mL, respectively. The group receiving 0.002 mg/animal total mAbs (Group 10, Table 1) had a NAC below the LLOQ.

**Figure 2:**
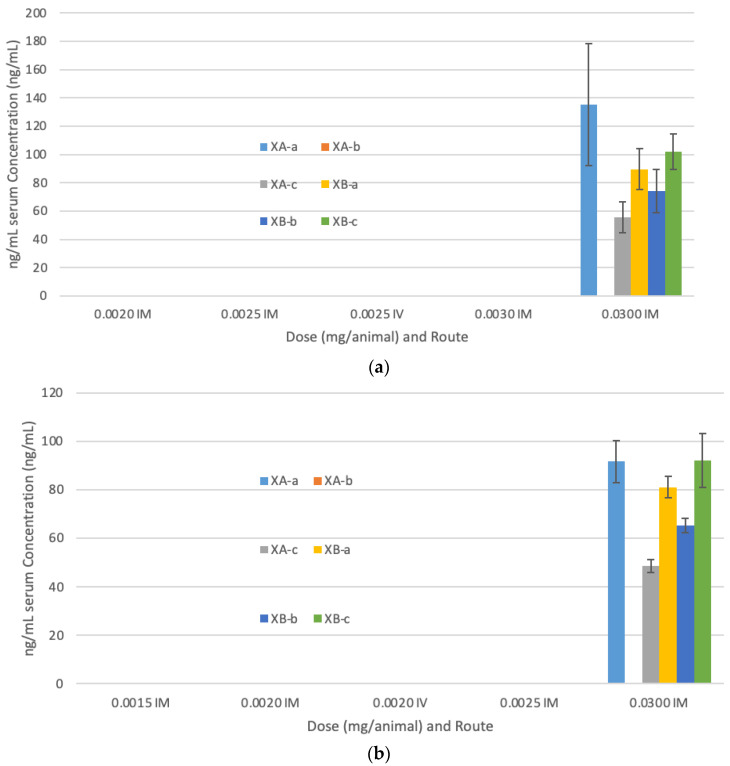
Serum concentrations of antibodies for the second challenge study. (**a**) BoNT A1 challenge animals; (**b**) BoNT B1 challenge animals. Lower limit of quantitation (LLOQ) for XA-a, XB-a, XB-b, and XB-c was 10 ng/mL; and for XA-c was 2.5 ng/mL; for XA-b was 200 ng/mL. Mean levels of antibodies ± standard deviation are shown. Serum samples were taken prior to dosing to ensure the antibodies were indeed in circulation in the animals prior to challenge.

For the second challenge study, we investigated the lower limits of dosing that provided protection. Serum concentrations of each antibody are shown in Figure 2. The MNAs from guinea pigs treated IM with a total of G03-52-01 of 0.03 mg/animal (Group 2, Table 2) resulted in a geometric mean NAC of 0.15 U/mL for serotype A. None of the other guinea groups in the serotype A cohort had a NAC above the LLOQ. The MNAs from guinea pigs treated IM with 0.002, 0.0025, or 0.03 mg total mAb/animal (Groups 5, 4 and 3, Table 2) or IV with 0.0025 mg total mAb/animal (Group 6) resulted in NACs below the LLOQ for serotype A. For serotype B, guinea pigs treated IM with 0.002, 0.025, or 0.03 mg of mAbs/animal (Groups 10, 9 and 8, Table 2) resulted in at least two of the three animals per group with NACs greater than the LLOQ with a geometric mean NAC of 0.015, 0.01 and 0.38 U/mL, respectively, per group. The MNAs from guinea pigs treated IM with 0.0015 mg of mAb/animal (Group 11, Table 2) resulted in two of the three NAC values below the LLOQ for serotype B. All three guinea pigs treated IV with 0.002 mg mAb/animal (Group 12, Table 2) had a NAC with a geometric mean of 0.014 U/mL. Animals had higher NAC values for antibodies against BoNT/B1 than against BoNT/A1, consistent with previous studies [38].

### 2.2. G03-52-01 Protects Guinea Pigs against Lethal Aerosol Challenge with BoNT/A and BoNT/B

Two inhalational BoNT/A1 and BoNT/B1 challenge studies were performed. Animals were divided into four separate runs per day of the aerosol challenge. A challenge dose of 100 GPLD_50_ for BoNT/A1 or BoNT/B1 was targeted for each study. The total inhaled dose was calculated from the amount of BoNT measured in the aerosol in GPLD_50_ and the measured total accumulated tidal volumes (TATV) in liters, as shown in Table 3.

For Study 1, animals in Run 1 received 27 GPLD_50_s (4329 mouse intraperitoneal LD_50_s, MIPLD_50_) and animals in Run 2 received 31 GPLD_50_s (4863 MIPLD_50_s) of BoNT/A1. For BoNT/B1, animals received 46 GPLD_50_s (9267 MIPLD_50_s) in Run 3 and 37 GPLD_50_s (7435 MIPLD_50_s) in Run 4 (Table 3). Prior to Study 2, the aerosol system was tested to verify the delivery efficiency and spray factors for aerosolizing BoNT/A1 and BoNT/B1. The target nebulizer concentrations were increased for Study 2 based on the results of the aerosol system verification. Therefore, for Study 2, animals in Run 1 and Run 2 received 169 GPLD_50_s (26,759 MIPLD_50_s) and 238 GPLD_50_s (37,626 MIPLD_50_s) of BoNT/A1 and 191 GPLD_50_s (38,203 MIPLD_50_s) and 180 GPLD_50_s (35,915 MIPLD_50_s) of BoNT/B1 for Runs 3 and 4, respectively.

For each challenge study, varying amounts of G03-52-01 were administered IM 48 h pre-challenge (Table 3). All animals dosed with PBS died within 15 h post-challenge with either BoNT/A1 or BoNT/B1 (Table 4). In the first challenge study, all of the animals administered more than 0.003 mg/animal of G03-52-01 survived for 14 days (the study duration) post-challenge with up to 31 guinea pig LD_50_ of BoNT/A. Only one animal died having received a similar BoNT/A challenge dose when given 0.002 mg/animal G03-52-01. None of the animals treated IM with a total G03-52-01 concentration of 0.03, 0.006, or 0.003 mg G03-52-01 exhibited clinical signs of botulinum intoxication through Day 14 post-challenge. Nine of ten animals treated IM with the lowest total anti-BoNT/A/B concentration of 0.002 mg/animal (Group 14) and five of the six animals that received vehicle IM and were challenged with BoNT/A1 exhibited clinical signs prior to death or euthanasia. One animal treated with 0.002 mg/animal of G03-52-01 did not exhibit clinical signs of botulinum intoxication throughout the 14 day post-challenge observation period. Clinical signs of botulinum intoxication included lethargy, nasal discharge, rough hair coat, respiratory distress, prostration, and moribund status.

All animals that were dosed with 0.006 mg mAb/animal and higher survived challenge with up to 46 guinea pig LD_50_ of BoNT/B. Nine of ten animals that received a G03-52-01 dose of 0.002 mg/animal survived for the duration of the 14 day post-challenge observation, and five of ten animals treated IM with the lowest total G03-52-01 concentration of 0.002 mg/animal died. In comparison, five animals survived for 14 days post-challenge. For animals challenged with BoNT/B1, all animals treated IM with a total G03-52-01 concentration of 0.03 or 0.006 mg did not exhibit clinical signs of botulinum intoxication through Day 14 post-challenge. One of ten animals treated IM with a total G03-52-01 concentration of 0.003 mg exhibited clinical signs of botulinum intoxication prior to being euthanized 222.2 h post-BoNT/B1 challenge. Five of ten animals treated IM with the lowest total G03-52-01 concentration of 0.002 mg and four of the six animals that received PBS control IM and were challenged with BoNT/B1 exhibited clinical signs prior to death or euthanasia. Clinical signs of botulinum intoxication included lethargy, rough hair coat, salivation, nasal discharge, hunched posture, respiratory distress, ataxia, prostrate, and moribund status.

The second challenge study was conducted to identify the levels of protection G03-52-01 has against higher doses of BoNT/A1 and BoNT/B1 challenge following a similar dosing regimen. As shown in Table 4, only animals dosed with 0.03 mg/animal G03-52-01 survived challenge with up to 238 GPLD_50_s BoNT/A. Animals dosed with less G03-52-01 did not survive longer than 53.6 h post-challenge. However, time to death was prolonged in all other groups compared to the PBS controls (Table 4). Antibody delivery via IV was more protective than via IM. A 0.0025 mg dose of G03-52-01 administered IV allowed the animals to survive longer after BoNT/A1 challenge than animals administered the same dose IM (42.9 h compared to 28.6 h). None of the animals treated IM with a total G03-52-01 concentration of 0.03 mg and challenged with BoNT/A1 exhibited clinical signs of botulinum intoxication through Day 14 post-challenge. All ten animals treated IM with 0.003 mg G03-52-01 exhibited clinical signs. Nine of ten animals treated IM with a total G03-52-01 concentration of 0.0025 or 0.002 mg and eight of ten animals treated IV with 0.0025 mg G03-52-01 exhibited clinical signs prior to death. Four of the five animals that received PBS control IM died prior to the first observation period and one animal exhibited clinical signs prior to euthanasia. Clinical signs of botulinum intoxication included lethargy, nasal discharge, eye discharge, rough hair coat, salivation, respiratory distress, ataxia, prostrate, and moribund status.

None of the animals dosed with less than 0.0025 mg G03-52-01 survived a challenge of 191 GPLD_50_s of BoNT/B1. Only two animals survived for 14 days after challenge with BoNT/B1 when administered 0.0025 mg mAb/animal, while all animals survived for 14 days after receiving 0.03 mg mAb/animal 48 h before toxin challenge. Similarly, animals administered 0.002 mg mAb/animal IV survived longer than those administered a similar mAb dose IM when challenged with BoNT/B1 (67.29 h compared to 58.1 h). None of the animals treated IM with a total G03-52-01 concentration of 0.03 mg and challenged with BoNT/B1 exhibited clinical signs of botulinum intoxication through Day 14 post-challenge. Two of ten animals treated IM with 0.0025 mg G03-52-01 did not exhibit or exhibited mild clinical signs of botulinum intoxication and survived through Day 14 post-challenge. Nine of ten animals treated IM with 0.002 and 0.0015 mg G03-52-01, all ten animals treated IM with 0.003 mg or IV with 0.002 mg G03-52-01, and the six animals that received PBS IM exhibited clinical signs of botulinum intoxication prior to death or euthanasia. Clinical signs of botulinum intoxication included salivation, hunched posture, nasal discharge, lethargy, rough hair coat, eye discharge, prostrate, ataxia, respiratory distress, prolapsed rectum, and moribund status.

## 3. Discussion

Botulism is a neuroparalytic syndrome that can progress to respiratory malfunction and death. Rapid diagnosis and treatment are essential to control botulism progression. A recombinant vaccine is currently under development (rBV A/B) to protect the warfighter from inhalational botulism due to BoNT/A1 and BoNT/B1 exposure [32,33]. A potential alternative to active vaccination is passive immunization achieved by administering a neutralizing antibody. Polyclonal antibodies, such as BIG-IV and equine BAT, have severe limitations that can be potentially overcome by recombinant human monoclonal antibodies [33]. Here we show that passive immunization with human or humanized mAb combinations is a viable alternative to active immunization to prevent botulism. This work expands our previous investigations with two different three antibody combinations [38]. We have co-formulated six antibodies to protect against BoNT/A and BoNT/B with a single cocktail of antibodies. In a single injection, we show here that the six-antibody cocktail can completely protect guinea pigs from an aerosol challenge of BoNT/A or BoNT/B challenge 48 h after administration. Protective levels of NAC were detected at doses of 0.03 mg/animal against BoNT/A and 0.002 mg/animal against BoNT/B, and 100% of the animals were completely protected against the BoNT challenge. 

The current studies’ design was based on previous PK results and the guinea pig model’s limitations for determining individual animal NACs before the BoNT challenge [38]. A first-in-human dose-escalation study evaluating the PK after administering increasing doses of anti-BoNT/A antibodies administered IV to humans resulted in a serum half-life of 15.5 to 26.9 days after a 0.33 mg/kg dose [39]. It is not unexpected for the observed short half-lives of human antibodies in guinea pigs due to an anti-drug antibody response observed between days 7 and 21 [38].

In the present studies, all animals that were administered vehicle only and were challenged with BoNT/A1 died between 7.8 and 14.3 h demonstrating that a lethal dose of BoNT was delivered. For the first challenge study, the efficacy of G03-52-01 was shown by the survival of all animals administered an IM dose above 0.003 mg/animal and challenged with 27 or 31 GPLD_50_s of BoNT/A1 through the 14 day post-challenge observation period. Animals that received 0.03 mg/animal of G03-52-01 had geometric mean NACs of 0.11 U/mL. The animals that were administered 0.003 or 0.006 mg/animal of G03-52-01 survived the BoNT/A1 challenge with geometric means below the LLOQ of the MNA. Even animals for which we could not detect NACs survived a lethal challenge of BoNT/A1, suggesting that very low levels of antibodies were protective, demonstrating the potency of these mAbs. However, ninety percent of the animals that were administered 0.002 mg/animal of G03-52-01 with a geometric mean below the LLOQ of the MNA died an average of 116.4 h post-challenge.

For the second challenge study, we next investigated whether the doses we are administering would protect against much higher doses of BoNT/A1 challenge. When the inhaled challenge dose of BoNT/A1 was increased to 169 or 238 GPLD_50_s, animals that received 0.03 mg/animal IM survived through the 14 day post-challenge observation period. These animals demonstrated a group geometric mean NAC of 0.15 U/mL, similar to that observed in the first study. All animals in the groups that received 0.0025 or 0.002 mg/animal of G03-52-01 IM demonstrated group mean NAC below the LLOQ of the MNA and died with an average time to death of 28.6 and 19.6 h, respectively. Although all of these animals did not survive the challenge, there appears to be a dose-dependent increase in time to death with the higher doses of G03-52-01 (Table 4). All animals treated with 0.0025 mg/animal IV and challenged with either 169 or 238 GPLD_50_s had a group geometric mean NAC below the LLOQ of the MNA and did not survive with an average time to death of 42.9 h.

Overall, for BoNT/A, animals that received G03-52-01 at a dose of 0.03 mg/animal had geometric mean NAC values above the LLOQ, and all of these animals survived the inhalation challenge. However, animals dosed with 0.006 and 0.003 mg/animal of G03-52-01 had geometric mean NACs below the LLOQ of the MNA. All of the animals in these groups survived the inhalation challenge with the lower BoNT/A1 concentration but were not protected when the BoNT/A1 concentration was increased. Administration of G03-52-01 below 0.003 mg/animal either IM or IV did not significantly protect guinea pigs against a BoNT/A1 inhalation challenge.

In the present studies, all animals that were administered vehicle only and were challenged with BoNT/B1 died between 10.6 and 11.5 h post-challenge, demonstrating that a lethal dose of BoNT was delivered. For the first challenge study, all animals treated with 0.03 or 0.006 mg/animal of G03-52-01 and challenged with 37 or 46 GPLD_50_s of BoNT/B1 survived through Day 14 post-challenge. The geometric mean NACs were 0.46 and 0.097 U/mL for the 0.03 and 0.006 mg/animal groups, respectively. Ninety percent of the animals that received 0.003 mg/animal of G03-52-01 survived the BoNT/B1 challenge and demonstrated a geometric mean NAC of 0.015 U/mL. The one animal that died in this group survived to 222.2 h post-challenge. Animals that received 0.002 mg/animal G03-52-01 had a 50% survival rate and a geometric mean NAC below the LLOQ.

For the second challenge study, animals that received 180 or 191 GPLD_50_s and 0.03 mg/animal of G03-52-01 IM survived through Day 14 post-challenge with a geometric mean NAC of 0.038 U/mL. In contrast, 80% of the animals treated with 0.0025 mg/animal of G03-52-01 with a geometric mean NAC of 0.01 U/mL died within 91.5 h post-challenge. All of the animals that received 0.0015 mg/animal of G03-52-01 died within 41.4 h post-challenge and demonstrated a geometric mean NAC below the LLOQ for BoNT/B1. For the groups with 80% to 100% mortality, the time to death increased as the G03-52-01 concentration increased for each of the BoNT/B1 challenge concentration (Table 4), indicating some level of protection even at this low dose.

This work furthers the evidence that passive immunization with human or humanized mAb combinations is a viable alternative to active immunization to prevent botulism [38]. In the present study, we combined NTM-1631 and NTM-1632 to create G03-52-01 and demonstrate that this combination will completely protect guinea pigs against lethal aerosol doses of BoNT/A1 and BoNT/B1 challenge 48 h after mAb administration at the high dose administration. Complete protection was also observed in animals with geometric mean NACs below the LLOQ when challenged with the lower doses of BoNT/A1 (27 or 31 GPLD_50_s) and BoNT/B1 (37 or 46 GPLD_50_s). This protection was observed at doses as low as 0.003 mg/animal for 100% protection against the lower doses of BoNT/A1 and 0.006 mg/animal for 100% protection against the lower doses of BoNT/B1. The protection we observed with the lower G03-52-01 levels against BoNT aerosol challenges is remarkable and confirms these antibodies’ potency. These doses of G03-52-01 were not protective against the higher doses of BoNT/A1 (169 or 238 GPLD_50_s) or BoNT/B1 (180 or 191 GPLD_50_s). While the NAC that protects humans against botulism is unknown, antibody titers likely to be protective in humans have been extrapolated from animal studies. Studies in guinea pigs indicate that serum antitoxin levels of 0.02 units/mL withstood challenge with 200,000 mouse LD_50_s of BoNT, and this value was set as the protective level in humans [40]. Separately, Fiock et al. [41] determined protective antibody titers in guinea pig at geometric mean NAC levels of 0.02 and 0.005 U/mL for BoNT/A1 and BoNT/B1, respectively. NTM-1631 and NTM-1632, which together make up G03-52-01, are significantly more potent than HBAT [38].

Although this study and our previous study [38] support future development of this drug candidate, there are limitations to these studies. First, guinea pigs are surrogate animal models. Second, these studies investigated only inhalation exposure to the BoNTs and not all of the various exposure routes. The inhalation route is a common route of administration and would be the most common route of administration in a terrorist attack using BoNT as a weapon. Future studies may also include a therapeutic evaluation of G03-52-01 and determine how long after exposure to the toxins that these antibodies still demonstrate efficacy. Regardless of the limitations of this study, the data presented herein demonstrate that this cocktail of antibodies can protect against aerosol challenges even at undetectable NAC levels.

The CDC recommended no further vaccine boosts for humans with neutralizing antibody titers greater than 0.25 IU/mL. Thus, human protection against botulism would be provided by BoNT antibody titers between 0.02 and 0.25 IU/mL for BoNT/A and 0.005 and 0.25 IU/mL for BoNT/B. Based on the ED50 of 40 IU/mL and Phase 1 clinical trial data on the NTM-1631 cocktail at an injected dose of 0.33 mg/kg, a peak serum titer of 94 IU/mL was observed, resulting in eight half-lives to reach 0.25 IU/mL and twelve half-lives to reach 0.022 IU/mL [39]. We observed 100% protection against intoxication with neutralizing antibody concentrations below the LLOQ, suggesting that this cocktail of antibodies can be extremely efficacious, even at lower doses. It is also possible that a longer duration of protection could be achieved by using a dose of G03-52-01 greater than 0.33 mg/kg.

## 4. Conclusions

In conclusion, we have demonstrated that a lyophilized six-mAb cocktail can prevent the development of botulism due to BoNT serotypes A and B after aerosol exposure in guinea pigs. We have demonstrated 100% survival of the guinea pigs from a lethal challenge of BoNT/A and BoNT/B, even when the geometric mean NAC is at the LLOQ. This result extends previous findings that the three-mAb single-serotype antitoxins for BoNT A and B protected guinea pigs from developing botulism after aerosol exposure. We postulate that the duration of protection in humans would be long lived based on mAb half-life and this combination’s potency. The results presented here that this antibody cocktail drug may be useful for both treatment and botulism prevention.

## 5. Materials and Methods

### 5.1. Monoclonal Antibodies

The oligoclonal mixture of six monoclonal antibodies (mAbs)—three against BoNT/A, and three against BoNT/B—G03-52-01, is comprised of an equimolar mixture of mAbs XA-a, XA-b, and XA-c, and mAbs XB-a, XB-b, and XB-c [39,42].

### 5.2. BoNT Challenge Material

The BoNT challenge materials were dilutions of the complex form of BoNT/A subtype A1 and BoNT/B subtype B1 purchased from Metabiologics (Madison, WI, USA). The BoNT/A1 was produced from a *C. botulinum* Hall A strain. The BoNT/B1 was produced from the *C. botulinum* Okra strain. The specific activity of the BoNT/A1 and BoNT/B1 challenge materials was 3.2 × 10^7^ and 7.5 × 10^7^ MIPLD_50_ U/mg protein, respectively [10]. The activity of BoNT for guinea pigs was previously measured at Battelle as one BoNT/A1 GPLD_50_ is equivalent to 158 MIPLD_50_s and one BoNT/B1 GPLD_50_ is equivalent 200 MIPLD_50_s (unpublished data). These measured values are in concordance with published values of BoNT/A and BoNT/B aerosol LD_50_s in guinea pigs (141 MIPLD_50_s for BoNT/A and 350 MIPLD_50_s for BoNT/B [43]. The research was conducted at a CDC Select Agent Program registered facility and the protocol was reviewed by the local Biosafety Committee.

### 5.3. Control Samples

The BoNT challenge materials and samples for the mouse neutralization assays (MNA) were diluted in 30 mM phosphate-buffered saline (PBS, pH 6.2) containing 0.2% (w/v) gelatin. All aerosol samples were collected in PBS, pH 7.2. The guinea pigs in the animal challenge studies received sterile phosphate-buffered saline (PBS), pH 6.5.

### 5.4. Pharmacokinetics

Animals were studied in five cohorts—PBS, G03-52-01 at doses of 0.03, 0.006, 0.003, and 0.002 total mg IM—in the first study. The second study consisted of six cohorts—PBS, G03-52-01 at doses of 0.002, 0.0025, 0.003, 0.03 total mg IM and 0.0025 total mg IV—when challenged with BoNT/A1 and six cohorts—PBS, 0.03, 0.0025, 00.2, 0.0015 total mg IM and 0.002 total mg IV—when challenged with BoNT/B1. Measurement of antibody circulating concentration post-IM or -IV dosing was performed using guinea pig serum collected from three animals in each treatment group before the BoNT aerosol challenge. Anti-BoNT/A antibodies were assessed using an enzyme-linked immunosorbent assay (ELISA). The levels of circulating anti-BoNT/B antibodies were assessed using an electrochemiluminescence (ECL) assay. The amount of toxin neutralization afforded by the antibodies (neutralizing antibody concentration, NAC) was assessed separately for each of the eight cohorts using the MNA with BoNT/A1 and BoNT/B1 with concomitant mAb concentration measurements as described below. 

#### 5.4.1. Electrochemiluminescence

The method to measure mAb concentration in guinea pig serum is based on a bridging immunoassay using Meso Scale Discovery (MSD) electrochemiluminescence (ECL) format [39]. The antibody specific domains used are recombinant domains of BoNT A or B [42,44]. Biotinylated and ruthenylated domains were used as the capturing and detecting reagents for the assay, respectively. The assay uses the bivalent binding capability of the IgG to form a bridging complex with the biotinylated domain and the ruthenylated domain to generate ECL signals for the measurement of the target antibody concentration in serum. Six assays were developed using the same format for each antibody assay [39] as described previously [38].

#### 5.4.2. ELISA

Three separate enzyme-linked immunosorbent assay (ELISA) methods were used to measure concentrations of the BoNT/A monoclonal antibodies—XA-a, XA-b, and XA-c—in guinea pig serum. 

In the XA-a, XA-b, and XA-c assay methods, the domains were coated at concentrations of 1.0, 20.0, and 5.0 µg/mL [42,44]. Nunc Maxisorp 96-well plates (ThermoFisher Scientic, Waltham, MA, USA) are used in each assay. After a coat incubation period of 12–24 h, each plate was washed and blocked for 1–4 h. Standards, controls, and samples were diluted at the minimum required dilution of 1:20, with some samples being further diluted into the standard curve range. Diluted standards, controls, and samples were added to the coated and blocked plate for three hours at room temperature on a shaker. During sample incubation, the XA-a, XA-b, and XA-c antibodies in guinea pig serum bind with the coated domains. The plate is then washed and goat anti-human IgG (H + L)-HRP was added to the plate and incubated for one additional hour. The plate was washed, and 3,3′,5,5′-tetramethylbenzidine (TMB) substrate solution was added to the plate and incubated in the dark for approximately 20 min before the addition of stop solution. The plate was read on a microplate reader at 450 nm for detection and 630 nm for reference.

A calibration curve was generated from the resulting absorbance values and plotted using a four-parameter logistic curve fit using 1/y^2^ weighting. The concentrations of each analyte in the QCs and study samples were interpolated from the calibration curve generated.

### 5.5. Measurement of Neutralizing Antibody Concentration (NAC)

NACs were determined using a standardized and validated mouse neutralization assay, as described previously [38]. NACs were determined using a standardized and validated MNA based on methods developed by Cardella and Hatheway and Dang [43,45]. Battelle Biomedical Research Center (Columbus, OH, USA) provides the only FDA approved, statistically validated MNA for determining anti-BoNT antibody concentrations (NACs) in the United States [46]. The NACs in this study were determined by Battelle using identical procedures and reference standards as those used for NAC determinations in the development of BIG-IV and its eventual licensure in 2003 [46,47] and as reported in other publications [46,47]. Male CD-1 (ICR) mice (*Mus musculus*) purchased from the Charles River Laboratory and weighing 17 to 23 g were used for the MNA. A standard curve assay containing nine square-root of two dilutions of antitoxin reference standards was prepared for each set of guinea pig serum samples tested. The reference standards were PI-A, Botulinum Immune Globulin, F(ab’)_2_, Heptavalent, Equine, Lot AAT manufactured by PerImmune, Inc. (Rockville, MD, USA) and calibrated in Units (1 U = 10,000 mouse IP LD_50_s/mL for BoNT/A1 and WHO-B, Lot 13209 3000 prepared and characterized by the Statens Serum Institut (Copenhagen, Denmark) and expressed in IU/mL (where IU = 10,000 mouse IP LD_50_s/mL) for BoNT/B1. Eight 4-fold dilutions were prepared for each serum sample. The antitoxin reference standards and guinea pig serum samples were titrated against a fixed amount of BoNT (44 mouse LD_50_/mL for BoNT/A1 and 25 mouse LD_50_/mL for BoNT/B1), incubated for 60 to 120 min at room temperature and 0.2 mL was injected IP into six mice per dilution. The BoNT/A1 and BoNT/B1 challenge concentrations were based on calibration experiments performed to determine the concentration that was 50% neutralized by 0.02 U/mL of the PerImmune-A and 0.005 U/mL WHO-B antitoxin standards, respectively. Survival was measured over 96 ± 2 h post-injection. To determine the ED_50_ for the antitoxin standards and serum samples, a probit dose–response curve was fitted to the lethality results as a function of the base 10 logarithm of antibody concentration or serum sample dilution. The NAC was calculated as the ratio of the ED_50_ of the standard curve, over the ED_50_ of the test curve.

### 5.6. Animal Challenge Studies

The research was conducted in compliance with the Animal Welfare Act (AWA, 7 USC §2131, 2002, 2007 and 2008) and other federal statutes and regulations relating to animals and experiments involving animals and adhered to the principles stated in the Guide for the Care and Use of Laboratory Animals (Battelle Biomedical Research Center Protocol Number 4298, approved 22 March 2018). All animal procedures were conducted under protocols approved by the Institutional Animal Care and Use Committees (IACUC) of Battelle Biomedical Research Center, in accordance with IACUC guidelines, https://www.nal.usda.gov/awic/ institutional-animal-care-and-use-committees. General procedures for animal care and housing were in accordance with the Association for Assessment and Accreditation of Laboratory Animal Care International (AAALAC) recommendations.

For the first aerosol challenge study, a total of 122 male Crl:( HA)Br guinea pigs (*Cavia porcellus*) were used. For the second aerosol challenge study, a total of 148 male Crl (HA)Br guinea pigs (*Cavia porcellus*) were used. Animals were randomized into one of ten treatment groups for the first study and one of twelve groups for the second study groups. Each animal was weighed prior to each study. The surviving animals were weighed at the end of the study (Day 14). Weight gain was determined by subtracting the beginning weight from the final weight. Average weight gain for each group was determined by averaging the weight gains from the total number of animals that survived till Day 14.

Two separate experiments were executed with different LD_50_s of BoNT. In each study, each animal received 0.3 mL IM of PBS or G03-52-01. The BoNT aerosol challenges were performed approximately 48 h after antibody/PBS administration with calculation of challenge doses summarized in Table 2. In Study 1 aerosol challenge experiment, animals were given 27 LD_50_s or 31 LD_50_s of BoNT/A1, or a dose of 46 LD_50_s, or 37 LD_50_s of BoNT/B1. The Study 2 aerosol challenge experiment involved giving the animals a dose of 169 LD_50_s or 238 LD_50_s of BoNT/A1, or a dose of either 191 GPLD_50_s or 180 GPLD_50_s of BoNT/B1. Serum was collected from three animals in each treatment/challenge group on the day of the aerosol challenge to determine the NAC at the time of BoNT challenge using the MNA.

### 5.7. Aerosol Exposure System

Guinea pigs were challenged with BoNT delivered by inhalation using an aerosol delivery system as described previously [10,38,48]. The target doses were prepared for challenge by thawing the vials of BoNT and then diluting to the target nebulizer concentration in sterile 30 mM phosphate buffer (pH 6.1–6.3) containing 0.2% gelatin. A nose-only aerosol exposure system (CH Technologies Tower) was utilized for the aerosol challenges. The guinea pigs were loaded into constraint tubes that connect to the tower and were exposed, nose-only, for 12 min. The aerosol system works by forcing air into the system through high efficiency particulate air (HEPA) filters and then divided into a continuous air stream and an air stream that either flows into the Collision nebulizer (during aerosol generation) or by-passes it between aerosol generations. The BoNT aerosol, created by the nebulizer, was mixed with continuous air before being delivered to the exposure chamber. The aerosol was sampled for concentration dose determination of BoNT using an impinger (Model 7541, Ace Glass, Inc., Vineland, NJ, USA) chilled in an ice bath before and during sampling. The aerosol particle size distribution was measured using an aerodynamic particle sizer or inertial cascade impactor that was sampled from the exposure tower. The total inhaled dose for each animal was estimated from the aerosol concentration and animal weight. The average dose delivered via aerosol route was determined using the average of three MIPLD_50_ values for each impinger sample in conjunction with exposure parameters such as flow rate and length of exposure and estimates of minute volume using Guyton’s formula [49] and is reported as the estimated inhaled MIPLD_50_/animal.

### 5.8. Measurement of BoNT Challenge Concentrations

The mouse toxin potency assays were used to determine the BoNT concentration of the nebulizer and impinger samples in the MIPLD_50_/mL, as described previously [38].

### 5.9. Statistical Methods

All statistical analyses were performed on data for each iteration separately. For the efficacy experiments, mortality rates were tabulated for each experimental iteration, BoNT serotype, dose route, control or treatment, and G03-52-01 concentration. For each iteration, the survival proportion in each group treated with G03-52-01 was compared to that of the associated control group using the same dose route, using a two-sided Boschloo Test. For each BoNT in Study 2, the survival proportions in selected groups treated with G03-52-01 were compared across dose routes (IM versus IV) for two pairs of groups that received the same treatment dose. Probit dose–response models were fitted, as described previously [38].

For assays that failed due to excessive mortality, sample-specific lower limits of quantitation (LLOQs) were derived from hypothetical MNAs. The test curve just met the assay acceptance criteria and had the largest ED50 obtainable within the range of antibody concentrations tested. The observed standard curve ED50 (from the standard curve determined concurrently with the test sample) was also incorporated to allow for day-to-day variation in the assay’s performance. Thus, the LLOQ was calculated as the observed standard curve ED50 divided by the hypothetical test curve ED50. An adjusted NAC was then calculated with a value equal to one-half of the LLOQ for the test curve. Since the LLOQ varied based on the standard curve associated with each sample set, the resulting LLOQ value also varied slightly from one experiment date to the next.

Descriptive statistics (geometric means and 95% confidence intervals) for the adjusted NAC data were calculated for each experimental iteration, BoNT serotype, dose route, control or treatment, and treatment dose (G03-52-01 concentration). The total number of assays with resulting NACs above LLOQ were also tabulated. The calculated 95% confidence interval was reported for a geometric mean NAC only if at least two of the three MNAs produced a NAC above the LLOQ.

## Figures and Tables

**Table 1:** BoNT/A- and BoNT/B-neutralizing antibody concentrations (NAC)—first challenge experiment.

Treatment Group	Number of Animals	BoNT Serotype and Dose Route	Total Antibody Dose (mg) ^a^	Geometric Mean NAC (U/mL) ^b^ 95% Confidence Interval
1-Vehicle	6	A, IM.	0	LLOQ
2-Anti-BoNT/A/B	13	A, IM	0.030	0.11 (0.027, 0.43)
3-Anti-BoNT/A/B	13	A, IM	0.006	LLOQ
4-Anti-BoNT/A/B	13	A, IM	0.003	LLOQ
5-Anti-BoNT/A/B	13	A, IM	0.002	LLOQ
6-Vehicle	6	B, IM.	0	LLOQ
7-Anti-BoNT/A/B	13	B, IM	0.030	0.46 (0.30, 0.69)
8-Anti-BoNT/A/B	13	B, IM	0.006	0.097 (0.022, 0.44)
9-Anti-BoNT/A/B	13	B, IM	0.003	0.015 (0.0083, 0.028)
10-Anti-BoNT/A/B	13	B, IM	0.002	LLOQ

^a^ Animals were dosed with the designated levels of antibodies. ^b^ Serum was collected from three animals per group for NAC determination to demonstrate the NAC in the serum prior to challenge. The remaining ten animals went on the challenge timeline. Lower limit of quantitation (LLOQ).

**Table 2:** BoNT/A and BoNT/B neutralizing antibody concentration (NAC)—second challenge experiment.

Treatment Group	Number of Animals	BoNT Serotype and Dose Route	Total Dose (mg) ^a^	Geometric Mean NAC (U/mL) 95% Confidence Intervals ^b^
1-Vehicle	5	A, IM.	0	LLOQ
2-Anti-BoNT/A/B	13	A, IM	0.03	0.15 (0.016, 1.4)
3- Anti-BoNT/A/B	13	A, IM	0.003	LLOQ
4- Anti-BoNT/A/B	13	A, IM	0.0025	LLOQ
5- Anti-BoNT/A/B	13	A, IM	0.002	LLOQ
6- Anti-BoNT/A/B	13	A, IV	0.0025	LLOQ
7-Vehicle	5	B, IM.	0	LLOQ
8-Anti-BoNT/A/B	13	B, IM	0.03	0.38 (0.25, 0.057)
9- Anti-BoNT/A/B	13	B, IM	0.0025	0.01 (0.008, 0.013)
10- Anti-BoNT/A/B	13	B, IM	0.002	0.015 (0.0036, 0.63)
11- Anti-BoNT/A/B	13	B, IM	0.0015	LLOQ
12- Anti-BoNT/A/B	13	B, IV	0.002	0.014 (0.0044, 0.0046)

^a^ Animals were dosed with the designated levels of antibodies. ^b^ Serum was collected from three animals per group for NAC determination prior to challenging the animals. The remaining ten animals went on the challenge timeline.

**Table 3:** Summary of BoNT aerosol exposure parameters.

Parameters		Study 1		Study 2	
		BoNT/A1	BoNT/B1	BoNT/A1	BoNT/B1
Guinea pig LD_50_ delivered	Target	100	100	100	100
	Actual	27 (Run 1), 31 (Run 2)	46 (Run 3), 37 (Run 4)	169 (Run 1), 238 (Run 2)	191 (Run 3), 180 (run 4)
Inhaled dose (MIPLD_50_/animal) *	Average	4596	8351	32,131	37,059
Total accumulated tidal volume (TATV) (L)	Actual	2.20	2.20	2.17	2.19
Impinger concentration (MIPLD_50_/mL) *	Actual	1.39E + 04	2.34E + 03	2.26E + 04	3.54E + 04
Nebulizer suspension concentration (MIPLD_50_/mL) *	Average	3.22E + 06	5.81E + 05	4.52E + 06	7.51E + 06
Aerosol conc. (MIPLD_50_/L) *	Average	2.09E + 03	3.80E + 03	1.48E + 04	1.70E + 04
Mass median aerodynamic diameter (µm)	Actual	1.11	1.08	1.07	1.00
Exposure time (min)	Actual	12.00	12.00	12.00	12.00
Number of animals per exposure	Actual	20	20	20	20

* MIPLD_50_ = mouse intraperitoneal LD_50_s.

**Table 4:** Guinea pig challenge time to death and mortality.

Treatment Group	Total mAb Concentration (mg)	BoNT Challenge Serotype and Route	Average Time to Death (h)	Average Weight Gain (g) after 14 Days *	Mortality (Number Dead/Total Animals)
First Challenge Experiment					
1-PBS	0	A1	11.8	-	6/6
2-Anti-BoNT/A/B	0.03	A1, IM	N/A	145.4	0/10
3-Anti-BoNT/A/B	0.006	A1, IM	N/A	148.9	0/10
4-Anti-BoNT/A/B	0.003	A1, IM	N/A	91.1	0/10
5-Anti-BoNT/A/B	0.002	A1, IM	116.4	70.6	9/10
6-PBS	0	B1	11.5	-	6/6
7-Anti-BoNT/A/B	0.03	B1, IM	N/A	157.4	0/10
8-mAb/A/B	0.006	B1, IM.	N/A	156.9	0/10
9-mAb/A/B	0.003	B1, IM	222.2	135.8	1/10
10-Anti-BoNT/A/B	0.002	B1, IM	129.5	91.8	5/10
Second Challenge Experiment					
1-PBS	0	A1	7.8	-	5/5
2-Anti-BoNT/A/B	0.03	A1, IM.	N/A	131.1	0/10
3-Anti-BoNT/A/B	0.003	A1, IM	53.6	-	10/10
4-Anti-BoNT/A/B	0.0025	A1, IM	28.6	-	10/10
5-Anti-BoNT/A/B	0.002	A1, IM	19.6	-	10/10
6-Anti-BoNT/A/B	0.0025	A1, IV	42.9	-	10/10
7-PBS	0	B1	10.6	-	5/5
8-Anti-BoNT/A/B	0.03	B1, IM.	N/A	115.2	0/10
9-Anti-BoNT/A/B	0.0025	B1, IM	91.5	37.3	8/10
10-Anti-BoNT/A/B	0.002	B1, IM	58.1	-	10/10
11-Anti-BoNT/A/B	0.0015	B1, IM	41.4	-	10/10
12-Anti-BoNT/A/B	0.002	B1, IV	67.2	-	10/10

N/A indicates animals did not die during the experiment; * average weight gain for surviving animals.
